# Plasma ATP concentration and venous oxygen content in the forearm during dynamic handgrip exercise

**DOI:** 10.1186/1472-6793-9-24

**Published:** 2009-12-15

**Authors:** Rachel E Wood, Connie Wishart, Philip J Walker, Christopher D Askew, Ian B Stewart

**Affiliations:** 1 Institute of Health and Biomedical Innovation and School of Human Movement Studies, Queensland University of Technology, Queensland, Australia; 2 Department of Vascular Surgery, Royal Brisbane & Women's Hospital & University of Queensland, Queensland, Australia; 3 Faculty of Science, Health, and Education, University of the Sunshine Coast, Queensland, Australia

## Abstract

**Background:**

It has been proposed that adenosine triphosphate (ATP) released from red blood cells (RBCs) may contribute to the tight coupling between blood flow and oxygen demand in contracting skeletal muscle. To determine whether ATP may contribute to the vasodilatory response to exercise in the forearm, we measured arterialised and venous plasma ATP concentration and venous oxygen content in 10 healthy young males at rest, and at 30 and 180 seconds during dynamic handgrip exercise at 45% of maximum voluntary contraction (MVC).

**Results:**

Venous plasma ATP concentration was elevated above rest after 30 seconds of exercise (P < 0.05), and remained at this higher level 180 seconds into exercise (P < 0.05 versus rest). The increase in ATP was mirrored by a decrease in venous oxygen content. While there was no significant relationship between ATP concentration and venous oxygen content at 30 seconds of exercise, they were moderately and inversely correlated at 180 seconds of exercise (r = -0.651, P = 0.021). Arterial ATP concentration remained unchanged throughout exercise, resulting in an increase in the venous-arterial ATP difference.

**Conclusions:**

Collectively these results indicate that ATP in the plasma originated from the muscle microcirculation, and are consistent with the notion that deoxygenation of the blood perfusing the muscle acts as a stimulus for ATP release. That ATP concentration was elevated just 30 seconds after the onset of exercise also suggests that ATP may be a contributing factor to the blood flow response in the transition from rest to steady state exercise.

## Background

Despite over a century of inquiry, understanding of the mechanisms that achieve the close matching of oxygen supply to demand during exercise remains elusive. One mechanism that has strong logical appeal proposes a role for the red blood cell (RBC) in the regulation of vascular tone [1]. According to this hypothesis, the RBC releases adenosine triphosphate (ATP) in proportion to the number of unoccupied binding sites on the haemoglobin molecule in response to both low oxygen and mechanical deformation, which are characteristic of the microvasculature *in vivo*. The ATP then binds with purinergic receptors and effects a conducted vasodilation via an endothelium-dependent mechanism [2]. It has been shown using an isolated cell model that RBCs release ATP upon exposure to hypoxia [3] and mechanical deformation [4,5]. Furthermore, the intraluminal application of ATP in arterioles [6] and venules [7] results in local and conducted vasodilation. Despite some evidence to the contrary [8], findings in intact mammals during exercise also tend to support a role for RBC-released ATP in the regulation of vascular tone [9-11].

ATP concentration has been shown to increase in response to incremental exercise in the coronary circulation of dogs exercising on a treadmill [11] and in the femoral vein in humans during knee extensor exercise [9,10]. Consistent with studies using isolated RBC [3], the increase in ATP concentration was also strongly correlated with a decrease in venous oxygen (and carbon monoxide) fraction in humans (r^2 ^= 0.93-0.96) [10] and coronary venous oxygen saturation (r = 0.93) and coronary blood flow (r = 0.99) in dogs [11]. Furthermore, intra-arterial infusion of ATP results in a dose-dependent increase in vascular conductance in the forearm and leg [10,12,13], mimicking the response seen during exercise [10].

Currently the available data suggest a role for ATP in the "steady state" phase of exercise. The few studies that have measured ATP during exercise have done so at least 60 seconds into an exercise bout [8,10,11]. Whether ATP is also elevated earlier during exercise, prior to 60 seconds, is currently unknown. Given that the stimulus for ATP release, deoxygenation, is present as early as 10-12 seconds after the onset of contractions [14-17], it is reasonable to hypothesise that ATP may also be important early in exercise, as blood flow adapts to meet the demand of skeletal muscle during the rest to exercise transition. This would also be consistent with the recent description of the dynamic response characteristics of blood flow at the onset of forearm exercise being at least biphasic [18]. The first phase, beginning just 2 seconds after the onset of contractions, is most likely attributable to mechanical factors whereas the delayed onset (approximately 21 seconds) of the second phase is suggestive of negative feedback control as per the classical metabolic hypothesis [19].

The appearance and accumulation of possible vasodilatory substances should precede the onset of the vasodilation it is proposed to stimulate [20]. Accumulation of proposed vasodilators such as ATP released from RBCs would therefore be expected to precede the onset of second phase; that is, they should be present within approximately 30 seconds of the onset of contractions. As such, the present study was designed to test the following hypotheses: 1) ATP concentration will increase in the venous blood during dynamic handgrip exercise, 2) ATP concentration will be elevated just 30 seconds after the onset of exercise, and 3) the increase in ATP will correspond with a decrease in the oxygen content of the venous blood.

## Methods

### Participants

Ten healthy males (age 29 ± 6 years, height 178.9 ± 5.8 cm, and weight 76.9 ± 12.5 kg) volunteered to participate in this study after being provided with full written and verbal descriptions of the experimental procedures. All participants signed an informed consent form which was approved by the Human Research Ethics Committee of the Queensland University of Technology, Brisbane, Queensland, in accordance with the *Declaration of Helsinki*.

### Experimental design

**Figure 1:**
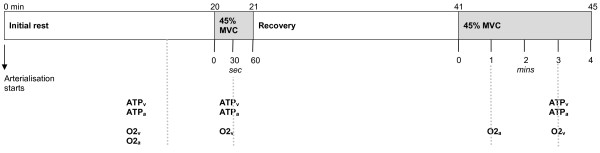
**Study design**. **ATP_v _**- venous blood sample for ATP assay, **ATP_a _**- arterialised blood sample for ATP assay, **O2_v _**- venous blood sample for measurement of blood variables, **O2_a _**- arterialised blood sample for measurement of blood variables. Heart rate was measured continuously throughout the session.

An overview of the experimental design is shown in Figure 1. Participants were asked to refrain from consuming caffeine, nicotine, and alcohol in the 12 hours prior to testing. Upon arrival at the laboratory, participants assumed a seated position and had venous catheters inserted in a retrograde direction into the median cubital vein of the exercising arm and in an antegrade direction into a superficial hand vein in the non-exercising arm. Following insertion of the catheters, participants rested in a seated position for 15 minutes with the exercising arm supported in a comfortable position on foam blocks and the hand of the non-exercising arm placed in a warm water bath to arterialise the venous blood [21]. Participants then completed two bouts of dynamic handgrip exercise at 45% MVC; the first was one minute in duration and the second was four minutes in duration. Twenty minutes of recovery was allowed between exercise bouts. Blood samples were drawn simultaneously from both arms at rest, and during each exercise bout for the determination of ATP concentration, and arterialised and venous oxygen saturation.

### Handgrip exercise

All handgrip exercise, which was undertaken using the dominant arm, was performed using a Digital Pinch/Grip Analyser (MIE Medical Research Ltd, Leeds, UK). The force produced by squeezing the device was measured by a Multi Analyser, and collected and analysed using the accompanying WinCAS software (MIE Medical Research Ltd, Leeds, UK).

### Maximum voluntary contraction (MVC)

Participants completed a maximum voluntary contraction (MVC) test for the determination of peak handgrip force. Each participant completed three, three-second trials, each separated by one minute of rest. MVC for each trial was taken as the peak force exerted during the three seconds. The MVC recorded for each participant was the average of three trials.

### Submaximal exercise bouts

All handgrip exercise was performed in the seated position, with the forearm slightly below the level of the heart to facilitate blood sampling. The handgrip dynamometer was supported on high density foam in a comfortable position below the level of the heart for each participant. Handgrip exercise involved squeezing a handgrip dynamometer in time with a metronome with a contraction frequency of 20 contraction cycles per minute. Each cycle consisted of a one-second contraction followed by two seconds of rest, a duty cycle that has been used previously [22-24]. The force production for each contraction was displayed in front of the participant on a visual scale. A target zone (± 5% of the target force) was marked in green on the scale and participants were asked to aim for this region with each contraction. Exerting a force on the handgrip dynamometer moved a vertical bar which turned from red to green when it was within the target zone. Participants were able to reproduce the required force with each contraction with a mean coefficient of variation of 6 ± 2% calculated from individual data. Peak force for each contraction was determined using custom-designed peak detection software. Peaks were then averaged to give the mean force for the duration of the exercise bout.

### Blood sampling and analysis

#### Cannulation

Upon arrival at the laboratory, participants had venous catheters (BD Insyte, 20 gauge, 30 mm, Becton Dickinson) inserted into a hand vein in the non-exercising arm and a forearm vein in the exercising arm. Catheters were connected to extension tubing (25 cm, 0.25 mL capacity, Terumo) and a three-way tap (BD Connecta, Becton Dickinson). In the exercising arm, the catheter was inserted into the median cubital vein and the entire length (30 mm) was advanced in a retrograde direction to enable sampling of deep venous blood. Blood samples drawn from catheters inserted in a retrograde direction into the median cubital vein have been shown to have oxygen saturations of ≤60% [25-27], reflective of blood from the skeletal muscle bed with little contribution from superficial veins [28]. The other catheter was inserted in an antegrade fashion into a superficial vein in the hand of the non-exercising arm.

#### Arterialisation

Venous blood from a hand vein in the non-exercising arm was arterialised, to provide a surrogate measure of arterial blood gases [21] and ATP concentration. Following cannulation, the hand of the non-exercising arm was placed in a warm water bath which was maintained at 45°C. The participant wore a waterproof glove to protect the skin and catheter from the water.

### Blood sampling protocol

Blood samples were drawn simultaneously from the exercising and non-exercising arms at rest, 30 seconds into the one-minute exercise bout, and 180 seconds into the four-minute bout. At each time point, a cuff was inflated at the wrist of the exercising forearm to minimise the contribution of the cutaneous circulation to the sample [29]. For each blood sampling time point, 3 mL of blood was first drawn into a syringe and discarded to ensure blood samples were not contaminated by residual blood or saline present in the catheter and extension tubing. Blood sampling for the assay of ATP, blood gases, and haemoglobin and haematocrit was performed according to the following procedure: 7 mL blood samples were drawn simultaneously from both arms and syringed into tubes precoated with EDTA (BD Vacutainer, K2E 10.8 mg Plus, Becton Dickinson), ~1 mL blood was drawn directly into a syringe from the exercising arm for the immediate measurement of blood gases, and a further 7 mL was drawn simultaneously from both arms and syringed into tubes precoated with EDTA for the later measurement of haemoglobin and haematocrit. Blood samples for the determination of arterialised blood gases from the non-exercising arm were drawn 120 seconds after those drawn from the exercising arm at rest, and 60 seconds into the four-minute exercise bout. Only one exercising arterialised sample was drawn considering arterial blood gases were not expected to change with small muscle mass exercise.

### Luciferin-luciferase assay

The samples of whole blood for the determination of ATP were transferred into microcentrifuge tubes and centrifuged immediately at 18 000 g for 60 seconds at 4°C to separate the plasma. Because ATP degrades rapidly in whole blood [30], care was taken to limit the time from drawing blood samples to centrifugation to approximately 150 seconds. Plasma was diluted 1 part in 100 in sterile, doubly distilled water. Pilot work in our laboratory showed that ATP in plasma is stable for at least 90 minutes when plasma is diluted in this manner. Diluted plasma was then assayed immediately using a commercially available firefly luminescent assay kit (FL-AA, Sigma, St Louis, Missouri) using an internal standard procedure, as recommended by Lundin [31]. All samples were assayed in duplicate. The coefficient of variation of nine duplicate resting plasma samples was 7%. Because ATP is released from the formed elements of the blood, plasma from each blood sample was assayed using the methods of Cripps [32] for plasma haemoglobin concentration.

### Analysis of blood gases

Arterialised and venous blood samples were analysed with a clinical blood gas analyser (i-STAT Portable Clinical Analyzer, Abbott Laboratories, Illinois, USA) using EG6+ cartridges for PO_2_, PCO_2_, SO_2_, pH, sodium, potassium, haemoglobin, and haematocrit. Arterial and venous oxygen content was calculated as: (O_2 _saturation × [Hb] × 1.39) + (0.003 × PO_2_). The i-STAT has been shown to measure P_a_O_2_, P_a_CO_2_, and pH with variability of 1.7, 1.2, and 0.6% on duplicate measurements, and measurements from the i-STAT agree well with those from the laboratory-based Radiometer ABL520 [33].

### Analysis and statistics

In order to detect a change in ATP concentration from ~600 to 1000 μM/L (~67% increase) with a power of 80% and p = 0.05, it was estimated that six participants would be required. All analyses were performed using SPSS 14. Data are mean ± SD unless otherwise indicated. Differences were considered significant if P < 0.05. Repeated measures ANOVA with Bonferroni correction for multiple comparisons with time (Rest, 30 seconds, and 180 seconds) as the within-subjects factor was used to determine whether ATP concentration and venous oxygen content changed in response to exercise. Relationships between ATP concentration and venous oxygen content at 30 seconds and at 180 seconds of exercise, and between the change in ATP concentration and change in venous oxygen content at 30 seconds and at 180 seconds of exercise were computed using Pearson product moment correlation coefficient. A 2 × 2 repeated measures ANOVA with time (Rest and 30 seconds of exercise) and position (arterial and venous) as within-subjects factors was used to test for differences in haemoglobin, haematocrit, PO_2_, PCO_2_, pH, sodium, and potassium.

## Results

### Force

Participants achieved a mean MVC of 448 ± 84 N and thus were required to work at 202 ± 38 N to achieve 45% MVC. The actual force produced was 212 ± 38 N, which corresponded to 48% MVC.

### Systemic response

Mean heart rate was 74 ± 10 bpm at rest and remained unchanged at 30 and 180 seconds of exercise (76 ± 8 and 75 ± 8 bpm, respectively; P > 0.05 for the main effect of time).

### ATP response

**Figure 2:**
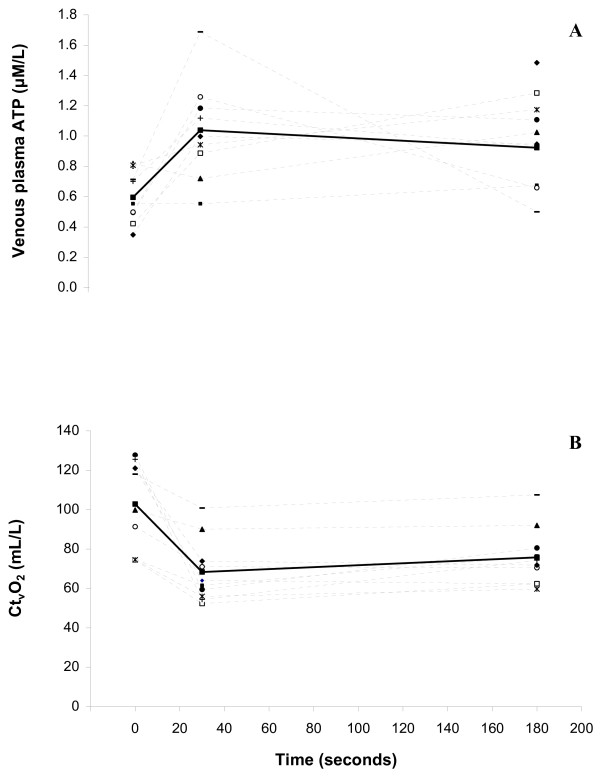
**ATP concentration (Panel A) and venous oxygen content (Ct_v_O_2_; Panel B) at rest, and 30 and 180 seconds into dynamic handgrip exercise at 45% MVC**. Dashed lines are the individual responses; n = 9 for ATP concentration and n = 10 for venous oxygen content; bolded solid line is the mean response (n = 9 for ATP concentration and n = 10 for Ct_v_O_2_).

Figure 2 (Panel A) shows the mean and individual ATP responses to exercise. Plasma ATP concentration was higher after 30 seconds of exercise compared to rest (1.04 ± 0.33 versus 0.60 ± 0.17 μM/L; P < 0.05) and remained elevated after 180 seconds of exercise (0.92 ± 0.26 μM/L; P < 0.05). ATP did not differ between the 30 and 180 second samples (P > 0.05). Arterialised ATP concentration was 0.79 ± 0.30 at rest, and remained unchanged with exercise (P > 0.05). These responses resulted in a tendency for a larger venous-arterialised ATP difference during exercise compared with rest, although this did not reach significance (P = 0.076).

### Blood oxygen content

There was a decrease in P_v_O_2_, S_v_O_2_, and Ct_v_O_2 _(P < 0.05 in all cases) from rest to 30 seconds of exercise, but no difference in the oxygenation of the blood between the 30 and 180 second time points during exercise (P > 0.05). Figure 2 (Panel B) shows the mean and individual venous oxygen content responses to exercise. Venous oxygen content decreased from 102.8 ± 22.5 mL/L at rest to 68.3 ± 16.0 mL/L after 30 seconds of exercise (P < 0.05), and remained lower than rest after 180 seconds of exercise (75.8 ± 14.8; P < 0.05). P_a_O_2_, S_a_O_2_, and Ct_a_O_2 _were unchanged in response to exercise (Table 1).

**Table 1:** Arterialised and venous haematocrit, haemoglobin, pH, ions, blood gases, and calculated oxygen content at rest, and 30 and 180 seconds into dynamic handgrip exercise at 45% MVC.

Variable		Rest	30 seconds	180 seconds
**Hct^b, e^**	**a**	0.42 ± 0.03	0.43 ± 0.03	
	**v**	0.42 ± 0.03	0.43 ± 0.03	0.43 ± 0.03
				
**Hb (g/L)^b, e^**	**a**	143 ± 9	146 ± 9	
	**v**	143 ± 10	145 ± 10	147 ± 11
				
**% SO_2_^a, b, c, d, e^**	**a**	95 ± 1	94 ± 2	
	**v**	52 ± 11	34 ± 7	38 ± 6
				
**CtO_2 _(mL/L)^a, b, c, d, e^**	**a**	182 ± 12	184 ± 12	
	**v**	100 ± 22	67 ± 16	75 ± 14
				
**PO_2 _(mmHg)^a, d, f^**	**a**	74 ± 5	71 ± 5	
	**v**	29 ± 5	23 ± 3	25 ± 2
				
**PCO_2 _(mmHg)^a, b, c, d, e, f^**	**a**	39 ± 3	41 ± 2	
	**v**	50 ± 3	57 ± 4	63 ± 5
				
**pH^a, b, d, e, f^**	**a**	7.42 ± 0.03	7.40 ± 0.02	
	**v**	7.37 ± 0.03	7.34 ± 0.02	7.31 ± 0.02
				
**K^+ ^(mmol/L)^a, b, c, d, e, f^**	**a**	4.0 ± 0.2	4.1 ± 0.2	
	**v**	4.0 ± 0.2	5.1 ± 0.5	4.7 ± 0.3
				
**Na^+ ^(mmol/L)**	**a**	139 ± 1	139 ± 1	
	**v**	139 ± 1	140 ± 1	140 ± 1

Data are mean ± SD. a - arterialised, v - venous, Hct - haematocrit, Hb - haemoglobin, K^+ ^- potassium, Na^+ ^- sodium, PO_2 _- partial pressure of oxygen, PCO_2 _- partial pressure of carbon dioxide, SO_2 _- saturation of haemoglobin with oxygen, CtO_2 _- oxygen content.

^a ^significant main effect for position; ^b ^significant main effect for time; ^c ^significant main effect for position by time interaction; computed from a 2 × 2 RM ANOVA (where time = rest and 30 seconds and position = arterial and venous), ^d ^30 second value significantly different from rest; ^e ^180 second value significantly different from rest; ^f ^180 second value significantly different from 30 second value; computed from a RM ANOVA for venous blood at rest, 30 seconds, and 180 seconds of exercise).

### Relationship between ATP concentration and blood oxygen content

Neither the correlation between ATP concentration and venous oxygen content nor the correlation between the change in ATP concentration and the change in venous oxygen content at 30 seconds of exercise was significant. At 180 seconds of exercise, there was a significant correlation between ATP concentration and venous oxygen content (r = -0.651, P = 0.021) and the change in ATP concentration was moderately correlated with change in venous oxygen content, although this relationship did not reach significance (r = 0.472, P = 0.084).

### Other blood variables

Table 1 contains the arterialised and venous haemoglobin concentration, haematocrit, oxygen saturation, oxygen tension, and oxygen content, pH, sodium, and potassium concentrations. During handgrip exercise, venous haematocrit and haemoglobin increased from rest, and this was significant by 180 seconds into exercise (P < 0.05). The pH of the venous blood was lower at both times points compared with rest (P < 0.05 in each case) and was also lower at 180 seconds compared with 30 seconds (P < 0.05). Arterialised pH did not change with exercise. Arterialised and venous sodium concentration, and arterialised potassium concentration remained unchanged in response to exercise. However, venous potassium concentration was higher than rest at 30 seconds of exercise (P < 0.05). Although the venous potassium concentration then decreased from 30 to 180 seconds (P < 0.05), it was still greater than rest at 180 seconds (P < 0.05).

## Discussion

The increase in ATP concentration during dynamic handgrip exercise is in agreement with early work in humans using mild static forearm contractions [34,35], and with more recent studies during knee extensor exercise in humans [10] and in dogs exercising on a treadmill [11]. A novel finding of the present study was that ATP concentration increased in the venous effluent of the forearm after just 30 seconds of dynamic handgrip exercise, and then remained at this higher level 180 seconds into exercise (Figure 2 Panel A). Previous studies have only measured ATP at least 60 seconds after the onset of contractions [9-11] and, as such, this is the first study to report an increase in ATP during the transition from rest to exercise. This finding is particularly interesting when considered in the context of the recently reported dynamic characteristics of the forearm blood flow response to exercise [18].

While blood flow has been measured for almost a century, the temporal aspects of the response have only recently been described [18,36,37]. The dynamic characteristics of blood flow at the onset of exercise are very similar in the arm and leg [18,36,38], and can be described as a second or third order response, depending on exercise intensity. The initial, rapid increase in blood flow starting approximately 2 seconds after the onset of contractions can be attributed to mechanical factors, such as the skeletal muscle pump [39] and contraction-induced vasodilation which occurs following a single muscle contraction [40]. The second phase begins at least 20 seconds after contractions start and continues until steady state is reached at lower intensities, or until the onset of a third phase at heavy intensities. The temporal characteristics of the second phase suggest that it may be under the control of negative feedback mechanisms, as per the classical concept of the metabolic hypothesis [19]. RBC-released ATP may have a role in this phase.

A primary stimulus for release of ATP from RBCs is the offloading of oxygen from the haemoglobin molecule [1,3,41-43]. There is an immediate increase in oxygen demand at the onset of muscle contractions that must initially be met by increasing oxygen extraction because blood flow has a mean response time of at least 40 seconds in the forearm when relatively heavy exercise is initiated from rest [18]. Thus there is a considerable lag from the onset of exercise until blood flow reaches levels commensurate to the new higher level of demand. The large increase in extraction of available oxygen is evidenced by a decrease in venous oxygen content just 10-20 seconds after the start of exercise in a deep forearm vein [14,44] and an increase in the deoxygenation signal from near-infrared spectroscopy ~12 seconds after exercise onset in the leg [15,16,45,46]. Oxygen extraction, at least in the exercising forearm below the level of the heart, also appears to reach its peak after just 60 seconds of exercise [14]. RBC-released ATP is believed to signal the mismatch between oxygen demand (metabolic rate of the muscle) and oxygen delivery (blood flow). Since blood flow takes at least 60 seconds to reach steady state [18], the stimulus for ATP release is potentially very large during the first 30 to 60 seconds of exercise. It is plausible that ATP plays a key role in the blood flow response at the onset of exercise. Previous suggestions that ATP is of primary importance in sustaining blood flow during steady state exercise may have come about because, until now, plasma ATP concentration had only been reported during the steady state [9-11].

### Relationship between ATP concentration and venous oxygen content

Although this study was not designed to verify the source of the ATP, the data are consistent with the release of ATP from the RBC. If, as the hypothesis suggests, ATP is released into the microcirculation in regions of high oxygen demand, ATP would be expected to increase to a greater extent in the local venous circulation than in the arterial circulation during exercise. This has been shown to be the case in the leg during knee extensor exercise [10] and was also the case in the present study in which the unchanged ATP concentration in the arterialised blood, and increased ATP in the venous blood (Figure 2), supports a net release of ATP from the microvascular bed in the forearm skeletal muscle.

ATP concentration has previously been shown to exhibit a strong inverse relationship with venous oxygen content during incremental exercise [10,11]. Since only a single intensity was used in this study, we cannot establish whether such a relationship exists in the forearm. However, at the intensity used in the present study (45% MVC), the increase in ATP in the exercising forearm corresponded with a decrease in deep venous oxygen content on a group level (Table 1, Figure 2). This also appears to be the case when the individual responses are considered (Figure 2 Panel A).

Venous oxygen content (Figure 2 Panel B) followed the same pattern in each of the ten participants, and seven of the ten participants exhibited a similar pattern of change in ATP concentration, indicating a consistency in the response. However, ATP concentration was not related to venous oxygen content at 30 seconds of exercise, nor was there a relationship between the change in venous oxygen content and the change in ATP concentration from rest to 30 seconds of exercise. The relationship between ATP concentration and venous oxygen content was stronger 180 seconds into exercise, where venous oxygen content could explain 42% of the variance in ATP concentration. Although it did not reach statistical significance, the moderate correlation between the change in venous oxygen content and the change in ATP concentration at 180 seconds of exercise also suggests that the two are related; that is those participants with the greatest change in venous oxygen content also had the greatest change in ATP concentration. While this alone does not confirm that the RBC is the source of the ATP, nor that ATP is involved in the regulation of blood flow, it is a critical test of the hypothesis considering offloading of oxygen from haemoglobin is a primary stimulus for the release of ATP from RBCs.

That the relationship between ATP concentration and venous oxygen content was weaker at 30 seconds than at 180 seconds of exercise is not surprising. The later sampling time (180 seconds) represents a relatively stable haemodynamic period in which blood flow is proportional to the demand of skeletal muscle metabolism. In contrast, blood flow is in a transition phase at 30 seconds of exercise [18]. Furthermore, there are inter-individual differences in the kinetics of the blood flow response to exercise even within a relatively homogeneous group [18]. If the time course of blood flow adaptation differs between individuals, then it follows that the time course of factors that regulate blood flow probably also differ. In addition it is also possible that ATP release from RBCs contributes to blood flow regulation to varying degrees in different individuals. As highlighted in several recent reviews [2,47,48], the regulation of blood flow is determined by the interplay between multiple factors that may be more or less important at varying times from the onset of exercise to steady state. To compound this issue further, there appears to be considerable redundancy in the regulation of blood flow to the extent that the simultaneous inhibition of multiple known vasodilatory pathways has been shown to affect blood flow only minimally. The poor relationship at 30 seconds of exercise is therefore likely to be reflective of inter-individual variability in the timing and contribution of ATP to the blood flow response, rather than indicating that ATP release is not related to venous oxygen content during the rest to exercise transition.

### Limitations

Forearm blood flow was not measured in this study due to technical limitations of the available technique, venous occlusion strain gauge plethysmography. However, maximal values of ~44 mL/min/100 mL have been measured using this technique in our laboratory during handgrip exercise at 45% MVC in participants with similar characteristics to those in the present study flows of this magnitude approach the peak exercising blood flows reported in other studies using venous occlusion strain gauge plethysmography and Doppler ultrasound [18,24,49]. While the findings of this study demonstrate that ATP is increased at high intensities of handgrip exercise, it remains to be seen whether there is a graded increase in ATP concentration with increases in workload in the forearm. The existence of such a relationship is questionable given that oxygen extraction appears to be maximised at low workloads in the forearm [14].

Another limitation was the use of concentration alone to characterise the ATP response to exercise. Because ATP originates from the RBC, its release is flow-dependent. Thus concentration may underestimate the true extent of the ATP release, especially when comparing between conditions where blood flow varies greatly [10], such as rest and exercise in this study. Concentration is dependent not only on release; it represents the balance between release of ATP and its rate of binding with purinergic receptors, degradation by ectonucleotidases, or inhibition by negative feedback mechanisms. To achieve the initial increase in ATP concentration between rest and 30 seconds of exercise, release must have exceeded clearance. This is supported by the increase (not significant) in the venous-arterial difference in ATP concentration and is consistent with the decrease in venous oxygen content which provides the primary stimulus for ATP release (Figure 2, Panel B). That ATP concentration at 180 seconds of exercise remained elevated but did not increase further (Figure 2 Panel A) indicates that clearance of ATP at that point is equal to release. During steady state, there remains a considerable stimulus for ATP release since oxygen extraction remains high during exercise; but oxygen delivery via the circulation meets the oxygen demand of the muscle at this point. Thus no further increases in ATP are expected. The measurement of blood flow during exercise would have permitted the calculation of the rate of release of ATP, which may have provided a more complete picture of the magnitude of the ATP response to exercise.

## Conclusions

This study provides the first evidence that venous ATP concentration increases in the forearm during dynamic handgrip exercise, that this increase is mirrored by a decrease in venous oxygen content, and importantly that there is a moderate but significant relationship between ATP concentration and venous oxygen content 180 seconds into exercise. In addition a novel, and especially interesting finding, is that plasma ATP concentration had increased as early as 30 seconds after the onset of muscle contractions and then remained elevated at 180 seconds into exercise. This pattern of response is consistent with ATP contributing to the second phase of the blood flow response at the onset of exercise, in addition to its role in sustaining blood flow during steady state.

## Competing interests

The authors declare that they have no competing interests.

## Authors' contributions

REW conceived the project, undertook the data collection and analysis, and wrote the manuscript; CW was instrumental in the development of the ATP analysis method and was involved in the data collection; PJW participated in the study design, performed all cannulations, and provided critical feedback on the manuscript; CDA participated in the study design and provided critical feedback on the manuscript; IBS supervised all stages, was involved in data collection, and prepared the final manuscript. All authors read and approved the final manuscript.
